# Structural and Antioxidant Properties of Compounds Obtained from Fe^2+^ Chelation by Juglone and Two of Its Derivatives: DFT, QTAIM, and NBO Studies

**DOI:** 10.1155/2016/8636409

**Published:** 2016-09-28

**Authors:** Aymard Didier Tamafo Fouegue, Julius Numbonui Ghogomu, Désiré Bikélé Mama, Nyiang Kennet Nkungli, Elie Younang

**Affiliations:** ^1^Department of Chemistry, Faculty of Science, University of Dschang, P.O. Box 67, Dschang, Cameroon; ^2^Department of Chemistry, Faculty of Science, University of Douala, P.O. Box 24157, Douala, Cameroon; ^3^Department of Inorganic Chemistry, Faculty of Science, University of Yaoundé I, P.O. Box 812, Yaoundé, Cameroon

## Abstract

The chelating ability of juglone and two of its derivatives towards Fe^2+^ion and the antioxidant activity (AOA) of the resulting chelates and complexes (in the presence of H_2_O and CH_3_OH as ligands) in gas phase is reported via bond dissociation enthalpy, ionization potential, proton dissociation enthalpy, proton affinity, and electron transfer enthalpy. The DFT/B3LYP level of theory associated with the 6-31+G(d,p) and 6-31G(d) Pople-style basis sets on the atoms of the ligands and the central Fe(II), respectively, was used. Negative chelation free energies obtained revealed that juglone derivatives possessing the O-H substituent (L_2_) have the greatest ability to chelate Fe^2+^ ion. Apart from** 1B**, thermodynamic descriptors of the AOA showed that the direct hydrogen atom transfer is the preferred mechanism of the studied molecules. NBO analysis showed that the Fe-ligand bonds are all formed through metal to ligand charge transfer. QTAIM studies revealed that among all the Fe-ligand bonds, the O_1_-Fe bond of** 1A** is purely covalent. The aforementioned results show that the ligands can be used to fight against Fe(II) toxicity, thus preserving human health, and fight against the deterioration of industrial products. In addition, most of the complexes studied have shown a better AOA than their corresponding ligands.

## 1. Introduction

Juglone (5-hydroxy-1,4-naphthoquinone) is a phenolic allelochemical responsible for walnut allelopathy and the inhibitory effect of black walnut (*Juglans nigra*) in associated plant species [1, 2]. It is a natural product that has shown a multitude of properties that are deemed beneficial in the fields of medicine, farming and aquaculture [3]. In the field of medicine, juglone has been shown to possess good antifungal properties, which are similar to those of some commercially available antifungal agents [4, 5]. In addition, it has been shown that juglone could be a promising chemopreventive agent for human intestinal neoplasia [6]. Furthermore, its antitumour [7–11] and antibacterial properties [5, 12] have been reported. Some experimental and theoretical studies have also emphasized the ability of juglone and its derivatives to inhibit the degradation of cells and foods by offering resistance to oxygen and its reactive species (known as antioxidant activity (AOA)) [10, 13, 14].

Antioxidants are capable of chelating transition metal ions (especially Fe^2+^ and Cu^+^) leading to the formation of stable complexes, thereby preventing these metals from participating in free radical generation [15–18], but the mechanism has not been studied exhaustively. The production of free radicals can lead to lipid peroxidation, protein modification, and DNA damage. Among the transition metals, iron is the most dangerous lipid oxidation prooxidant due to its high reactivity. In fact, the ferrous state of iron accelerates lipid oxidation by breaking down hydrogen and lipid peroxides to reactive free radicals via the Fenton reaction [19], thus degrading the quality of foods and causing several diseases [20]. Antioxidants are well known radical scavengers that are largely used to fight against these negative effects of free radicals, via the above mentioned mechanism. Besides removal of the metals, metal-chelating compounds can also alter their redox potentials, rendering them inactive. The use of natural metal chelators instead of the synthetic counterparts which may present some toxicity problems should be encouraged [21]. Several experimental and theoretical studies have been devoted to the elucidation of the metal-chelation mechanism of AOA [19, 21–25].

**Figure 1:**
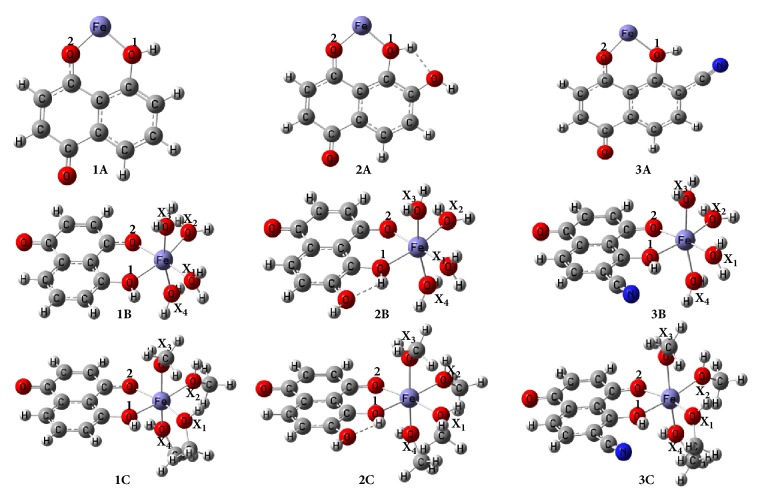
Optimized geometries of the studied molecules, obtained by applying the B3LYP/6-31G(d)(Fe)U6-31+G(d,p)(E) level of theory.

It has been demonstrated experimentally that deprotonated juglone has the capacity to chelate Fe^2+^ [13]. A Theoretical study to complement this experimental result is warranted. To the best of our knowledge, no such theoretical work exists in the literature. In this paper, we aim to investigate the Fe^2+^ chelating ability of neutral juglone and two of its derivatives (shown in Figure 1), as well as evaluate the AOA of the resulting compounds by the means of density functional theory (DFT) [26]. This work is therefore expected to contribute toward the development of new antioxidants, which has been the ambition that has attracted a great deal of researchers' attention in recent years, owing to the significance of antioxidants in biological processes as well as the processes in the food, pharmaceutical, and material industries. Specifically, the chelation of Fe^2+^ by three antioxidants in their neutral forms is studied at DFT/B3LYP level theory. The main antioxidant studied is juglone (L_1_), and the rest are its derivatives obtained by replacing the hydrogen atom* ortho* to the hydroxyl group of juglone either by OH (L_2_) or by CN (L_3_) groups, which are electron donating (EDG) and withdrawing (EWG) groups, respectively. The structural and electronic parameters of the resulting chelates or complexes have been analyzed, followed by the evaluation of the AOA of all compounds studied (Figure 1) by means of bond dissociation enthalpy (BDE), ionization potential (IP), proton dissociation enthalpy (PDE), proton affinity (PA) and electron transfer enthalpy (ETE) [17, 18]. Our main objective here is to study the effect of iron(II) chelation on the AOA of the three ligands (L_1_, L_2_, and L_3_).

## 2. Computational Details and Theoretical Background

### 2.1. Computational Details

All calculations were carried out using Gaussian 09W [27]. The input structures were prepared using the GaussView 5.0.8 program [28]. The DFT method which was designed especially for the study of coordination compounds [24] was associated with the B3LYP hybrid functional in this research endeavor [29]. In addition, DFT has been chosen because it has been used successfully to study radical scavenging activities of phenolic compounds [30–32]. Also, when compared to* ab initio* methods, DFT is very rapid and is often said to ally precision and the rapidity [33]. Faced with limited computational resources and large molecular sizes, we used a mixed basis set comprising the 6-31G^*∗*^ basis for the central metal ion and the 6-31+G^*∗∗*^ basis for every other element in the molecules studied. In addition, mixed basis sets have been recently employed for many studies on complexes and have been shown to speed up calculations, without altering the quality of theoretical results [21, 34, 35]. All computations on the closed shell systems were performed using the Restricted Kohn-Sham formalism while the Unrestricted Kohn-Sham formalism was adopted in open shell systems, in order to reduce spin contamination [36]. Ground-state geometries for all complexes have been fully optimized without any symmetry constraints. Vibrational frequency calculations have further been undertaken on the optimized geometries in order to confirm that the resulting equilibrium geometries were minima (no negative frequency) on the potential energy surface. Central metal-ligand charge transfer was evaluated by means of Natural Bond Orbital (NBO) analysis [37] as implemented in Gaussian 09. The Quantum Theory of Atom in Molecules (QTAIM) proposed by Bader [38] was used to evaluate the nature of all metal-ligand bonds, with the aim of determining their degree of covalency. QTAIM analysis was performed as implemented in multiwfn [39].

### 2.2. Theoretical Background

The chelates [FeL_*i*_]^2+^ (*i* = 1, 2 and 3) (Figure 1) optimized in this work are presumed to be formed according to

(1) $$\begin{array}{c} Fe^{2+}+L_{i}⟶{\left[\;FeL_{i}\;\right]}^{2+} \end{array}$$

The complexes [FeL_*i*_(X)_4_]^2+^, (*i* = 1, 2 and 3; and X = H_2_O or CH_3_OH) are presumed to be formed according to

(2) $$\begin{array}{c} Fe^{2+}+L_{i}+4X⟶{\left[\;{FeL}_{i}{\left(\;X\;\right)}_{4}\;\right]}^{2+} \end{array}$$

The use of water and methanol molecules as other ligands is because of the difficulty to find isolated form of Fe^2+^ and the abundance of such polar molecules in organisms [23].

The effect of multiplicity on the stability of all the nine chelates and complexes resulting from both (1) and (2) as presented in Figure 1 was first investigated. This was done via geometry optimizations of all the input structures in the singlet and quintet states based on the possible number of unpaired electrons of the central Fe^2+^ ion, in order to determine the geometry with the lowest ground-state energy. The binding energies (Δ*E*_int_), free energies (Δ*G*), and enthalpies (Δ*H*) of formation of the most stable geometries at standard conditions were calculated as follows:

(3) ΔEint=Ecomp−EL−4EX−EFe2+,

(4) ΔH=Hcomp−HL−4HX−HFe2+,

(5) ΔG=Gcomp−GL−4GX−GFe2+.

In these equations *E*, *H*, and *G* stand for the thermal energies, enthalpies, and free energies of formation of the respective species. Due to the absence of X ligands in the chelates, *E*_X_, *H*_X_, and *G*_X_ terms are nonexistent in their expressions for Δ*E*_int_, Δ*H*, and Δ*G*.

Using the most stable optimized geometry of each compound, the usual schemes of hydrogen transfer by antioxidants and the related parameters were calculated. These include the following.

*(i) The Direct Hydrogen Atom Transfer (HAT).* The direct hydrogen atom transfer (HAT) is the mechanism in which the phenolic H atom is transferred in one step by the antioxidant. BDE, which is the parameter used to evaluate HAT, is the reaction enthalpy for the mechanism. This parameter has been evaluated on all the hydroxyl groups of juglone and its two derivatives studied. The lower the BDE, the easier the dissociation of the phenolic O-H bond, as elucidated in (6) and (7) where X-H represents the ligands, chelates, or complexes and X^•^ is the radical compound resulting from the H atom abstraction:

(6) X-H⟶X•+H•

(7) BDE=HH•+HX•−HX-H

*(ii) The Sequential Electron Transfer Proton Transfer (SETPT)*. Here, an electron abstraction from X-H (characterized by the ionization potential (IP) of X-H) is followed by a proton transfer (characterized by the proton dissociation enthalpy (PDE) of the cationic radical X-H^+•^) as shown in the following:

(8) X-H⟶X-H+•+e−

(9) IP=HX-H+•+He−−HX-H

(10) X-H+•⟶X•+H+

(11) PDE=HX•+HH+−HX-H+•

*(iii) The Sequential Proton Loss Electron Transfer (SPLET)*. Here, a proton loss is followed by an electron transfer. The reaction enthalpy of the first step in the SPLET mechanism corresponds to the proton affinity (PA) of X^−^. The reaction enthalpy of the next step, the transfer of an electron by X^−^, is denoted as electron transfer enthalpy (ETE) and is calculated using the following equations:

(12) X-H⟶X−+H+

(13) PA=HX−+HH+−HX-H

(14) X−⟶X•+e−

(15) ETE=HX•+He−−HX−

The free energies of all aforementioned descriptors have also been calculated and denoted BDFE (bond dissociation free energy), IPFE (ionization potential free energy), PDFE (proton dissociation free energy), PAFE (proton affinity free energy), and ETFE (electron transfer free energy).

## 3. Results and Discussion

### 3.1. Effect of Multiplicity on Electronic Energies

Owing to the intrinsic relationship between spin multiplicity and ligand field, the effect of multiplicity on the total energies of the chelates and complexes studied (Figure 1) has been investigated. This was done in order to select the multiplicities of the molecules studied that correspond to their lowest energy (or most stable) geometries. In this regard, the singlet and quintet states corresponding to geometries in which the central Fe^2+^ ion has no unpaired electron and four unpaired electrons, respectively, were investigated for each chelate and complex. 〈*S* ^2^〉 values, calculated for all quintet states, are in the range of 6.0001–6.0006, which is close to the value of 6.000, corresponding to the pure quintet wave function. The total energies of the said states are displayed in Table 1. In this table, the complexes of L_1_ (juglone), L_2_ (the derivative with the OH group), and L_3_ (the derivative with the CN group) are denoted by** 1**,** 2**, and** 3**, respectively. The chelates are represented by the symbol** YA** (where **Y** = 1, 2 or 3). Similarly, the complexes containing the ligands H_2_O and MeOH are, respectively, represented by** YB** and** YC**.

It can be seen from Table 1 that the singlet state of** 1A** and** 1B** which are complexes of L_1_, are the most stable, whereas the quintet state is instead the most stable in the case of its MeOH containing complex (**1C**). It is obvious from the table that among the complexes of L_2_,** 2A** has the lowest energy singlet state, while** 2B** and** 2C** containing H_2_O and MeOH ligands, respectively, are constrained to the quintet state. The three complexes of L_3_ (**3A**,** 3B**, and** 3C**) are restricted to the quintet state. From the foregoing observations, the rest of this work has been limited to the singlet states of** 1A**,** 1B**, and** 2A** and the quintet states of** 1C**,** 2B**,** 2C**,** 3A**,** 3B**, and** 3C**.

### 3.2. Iron(II) Chelation Capacity of the Ligands

The capacity of juglone and its derivatives to chelate Fe(II) has been evaluated at 298.15 K and 1 atm via Gibbs free energy of formation calculations on the complexes, as shown in (5). The enthalpies of formation of the complexes as well as their binding energies were also calculated, and the results are presented in Table 2.

By inspection of Table 2 it can be seen that the free energies of formation of the three chelates (**1A**,** 2A**, and** 3A**) are negative, meaning that their formation is spontaneous. Among the chelates, the computed free energies increase in the order** 2A** <** 1A** <** 3A**, showing that L_2_ has the highest Fe^2+^ ion binding capacity and L_3_ has the lowest. This result is attributable to the fact that the presence of the EDG (the -OH group of L_2_) increases the electron density on the ligating oxygen atoms, thus increasing the metal-ligand interaction. The reverse is observed in presence of -CN (**3A**). The presence of H_2_O ligands decreases Δ*G* of formation of** 1A**, while the MeOH ligands rather result in its increment. In the case of** 2A**, the opposite of the previous observation is evidenced in Table 2, implying that while the presence of water ligands increases Δ*G* of formation of** 2A**, the presence of the methanol ligands instead leads to its reduction. The presence of H_2_O and MeOH ligands in** 3A** decreases its Δ*G* by 148 and 58 kJ/mol, respectively.

The negative values of the binding energies have proven that the complexes investigated are stable [25], corroborating the fact that their formation is thermodynamically feasible. The values of the enthalpies of formation of all complexes are also negative, showing that their formation is exothermic at 298.15 K and under 1 atm.

### 3.3. Geometric Parameters

This section is dedicated to Fe-ligand and O_1_-H bond lengths obtained from the optimized geometries of the complexes presented in Figure 1. It is clear from this figure that the chelates** 1A**,** 2A**, and** 3A** are perfectly planar, and the complexes** 2B**,** 2C**,** 3B**, and** 3C** are octahedral. The values of the bond lengths around the central metal are presented in Table 3.

The atomic numbering adopted in Table 3 is as presented in Figure 1. The length of the O_1_-Fe bond in each chelate increases in the order,** 2A** <** 1A** <** 3A**, which is the same order of their Δ*G* of formation. Hence, the values of Δ*G* and the O_1_-Fe bond lengths for the chelates are directly proportional. It can also be observed from Table 3 that the presence of H_2_O and MeOH ligands increases the values of the O_1_-Fe bond lengths in all the chelates. Some discrepancies between the O_1_-Fe bond lengths in** 1A**,** 2A**,** 3A** relative to those in the corresponding methanol complexes of 0.315, 0.358, and 0.225 Ǻ, respectively, have been observed.

The O_2_-Fe bond lengths have been found to be shorter than the O_1_-Fe counterparts in all chelates investigated. The lengths of the former have also been found to increase in presence of H_2_O and MeOH as ligands. Our results are in good agreement with those in the literature for similar studies on three naturally occurring phenolic antioxidants [23].

The lengths of all the X_*n*_-Fe bonds (*n* = 1, 2, 3 and 4; X = H_2_O or MeOH) are larger than 2 Ǻ. Among the complexes of juglone, that possessing MeOH ligands (**1C**) has the longest X_*n*_-Fe bond, and its formation is found to be the least spontaneous.

From inspection of the O_1_-H bonds of the three chelates, the ranking** 1A** <** 2A** <** 3A** can be made, showing that the bond is the longest in** 3A** with a length of 0.997 Ǻ. This is surprising because the O_1_-H group of** 2A** is involved in hydrogen bonding as depicted in Figure 1, which should elongate its O_1_-H bond rendering it the longest relative to those of the other chelates. Since the AOA is greatly influenced by the length of the phenolic O-H bond, these differences in O_1_-H bond lengths could have great consequences on the values of associated thermodynamic descriptors of AOA. Still from these findings, it can be predicted that among the chelates,** 1A** which has the shortest O_1_-H bond length should have the highest associated BDE value. The presence of the H_2_O and MeOH ligands is found to slightly decrease the length of the said bond. The presence of the methanol ligands has a more significant effect on the O_1_-H bond length of** 2A**. Indeed, the presence of the methanol ligand is found to reduce the length of the bond by 0.016 Ǻ. This is attributable to hydrogen bond strength reduction in** 2B**. The presence of the water ligands in** 2A** has virtually no effect on the length of the O_1_-H bond. The presence of both ligands individually reduces the length of the hydroxyl link of** 3A** by 0.011 and 0.013 Ǻ, respectively.

The lengths of the hydrogen bonds of** 1B**,** 2B**, and** 3B** are, respectively, 1.909, 1.920, and 1.961 Ǻ, which are in good agreement with those of the O_1_-H groups of the said molecules, since they are inversely proportional to the O_1_-H bond length. The lengths of the additional O-H bonds in** 2A**,** 2B**, and** 2C** (marked in Table 3 with *∗*) are shorter than those of O_1_-H groups, due to the fact that they are not engaged in any hydrogen bond.

### 3.4. Thermodynamic Descriptors of Antioxidant Properties

#### 3.4.1. HAT Mechanism

The calculated gas phase BDE values of the molecules investigated are presented in Table 4.

It is evident in Table 2 that the BDEs of the O_1_-H bond for the chelates increase in the order** 2A** <** 3A** <** 1A**. The fact that the BDE of O_1_-H is the lowest in** 2A** suggests that the hydrogen bond in which this group is engaged weakens the O_1_-H bond in this molecule. In addition, the phenoxy radical formed due to HAT by** 2A** is stabilized by an O-H⋯O_1_ hydrogen bond, further strengthening the AOA of** 2A**. The low BDE of** 3A** relative to that of** 1A** can be explained by the fact that its O_1_-H bond is longer and therefore weaker than that of** 1A**. From the foregoing observations, it can be concluded that the addition of the OH or CN groups to** 1A** reduces the BDE value of its hydroxyl group.

It is also clear from Table 4 that the BDE of the O_1_-H bond of juglone (L_1_) is lower than that of** 1A** by 191 kJ/mol, implying that Fe(II) chelation by juglone leads to an increase in the BDE of its O-H group. The presence of the water ligands in** 1B** results in a BDE value increment of 397 kJ/mol relative to that of** 1A**, which contains no H_2_O ligands. This can be attributed to the reduction in the O_1_-H bond length due to the presence of H_2_O ligands, as previously observed in Section 3.3. On the contrary, the introduction of the MeOH ligands into** 1A** decreases its BDE value.

It can be seen from Table 4 that the O_1_-H BDE of** 2A** is lower than that of L_2_ by 269 kJ/mol. Among the compounds studied,** 2A** has been found to exhibit the lowest O_1_-H BDE, showing that the HAT mechanism is most favorable in this molecule. Besides the electron donating ability of the hydroxyl substituent of** 2A**, the hydrogen bond in its phenoxy radical could have a great stabilizing effect that lowers its BDE value. The BDE value of** 2B** lies between those of** 2A** and L_2_. On the other hand the BDE value of** 2C** is much higher than those of both** 2A** and L_2_. Among the molecules investigated in this work, the highest BDE value (1240 kJ/mol) has been recorded for** 2C**. This is probably due to the fact that the hydrogen bond in** 2C** that involves the O_1_-H group is weaker than similar bonds in** 2A** and** 2B**. From these results it can be concluded that Fe(II) chelation by L_2_ decreases its BDE value, as opposed to the presence of MeOH ligands which greatly increases the BDE value.

Fe (II) chelation by L_3 _is found to decrease its BDE value by 128 kJ/mol. It has been found that the BDE values of** 3B** and** 3C** are lower than that of** 3A** by 35 and 49 kJ/mol, respectively. Generally, our results have shown that the BDEs of the O_1_-H bonds of the species currently investigated increases in the order** 2A** <** 1C** <** 3C** <** 3B** <** 2B** <** 3A** < L_2_< L_1_ < L_3_<** 1A** <** 1B** <** 2C**.

The BDE value of the additional O-H substituent in L_2_, designated by an asterisk (*∗*) in Table 3, is found to be lower than that of the O_1_-H bond, whereas the reverse is observed for its complexes. Therefore, the H atom of the O_1_-H bond in the complexes is more available for a radical attack.

The trend in the BDFE values of the molecules studied is similar to that of the BDE values. As a general observation, the BDFE values are found to be lower than the corresponding BDE values. The differences between the values of these descriptors are in the range 21–37 kJ/mol for the complexes studied. Furthermore, the BDFEs are all positive, signifying that the HAT mechanism is not spontaneous for all the complexes and ligands studied. These results are in good agreement with those found in the literature [23, 40].

#### 3.4.2. SETPT Mechanism

The first and the determining step in this mechanism is electron transfer which is characterized by the associated IP of the antioxidants. The calculated adiabatic IP values of the complexes and ligands are presented in Table 4. It is clear in Table 4 that Fe^2+^ chelation leads to an increase in the IPs of L_1_, L_2_, and L_3_. This result is in good agreement with some literature findings [21]. The IP values of the chelates have been found to increase in the order** 3A** <** 1A** <** 2A**. Surprisingly from this ranking,** 3A** possessing EWG exhibits the greatest electron transfer capability, while** 2A** containing EDG together with a hydrogen bond shows the least electron transferability.

While the presence of water ligands increases the IP of** 1A**, the MeOH ligands instead lead to its reduction. These ligands have been found to have a similar effect on the BDE of** 1A** as explained in Section 3.4.1. In the case of** 2A**, the opposite trend is observed wherein the presence of the water ligands instead leads to a reduction in its IP, whereas the MeOH ligands result in its increment. The introduction of either water or MeOH ligands reduces the IP value of** 3A** in which case the effect of the MeOH is found to be the greatest. The presence of these ligands similarly affects the BDE of** 3A** and its complexes.

Unlike BDFE, the values of IPFE for the complexes are generally found to be slightly higher than their IP, as shown in Table 4. The only exceptions to this trend are the IPFEs of** 1A** and** 2A** which are lower than IP (by 2 kJ/mol) and equal to IP, respectively.

The PDE values of the complexes displayed in Table 4 are all lower than those of the ligands. We have attributed this to the fact that the cation radicals of the complexes are less stable and are therefore more reactive than those of the ligands, as shown by the IP values presented in this table. The PDE values of the chelates are smaller than those of the free ligands as well as those of complexes. The PDE values of** 2A** (−539 kJ/mol) and** 3A** (−233 kJ/mol) are negative, which is an indication that the proton transfer process of their cation radicals is exothermic. Since the PDFE values of the chelates (**1A**,** 2A**, and** 3A**) are all negative, it can be concluded that the proton transfer process by their cation radicals is spontaneous, which is not the case for the rest of the compounds in Table 4.

#### 3.4.3. SPTET Mechanism

This mechanism begins with the transfer of a proton, designated as PA. This is followed by an electron transfer from the resulting anion, also designated as ETE. The values of PA and ETE for the ligands and their complexes as well as their free energies (PAFE and ETFE) calculated in this work are presented in Table 4. This table shows that, apart from** 2C**, the chelates and complexes have lower PA values than their respective ligands. Like the IP values, the PAs of the chelates can be classified in the order** 3A** <** 1A** <** 2A**. This can be explained by the fact that EWG (-CN) increases the acidity of the H atom of the hydroxyl group whereas EDG (-OH) reduces the hydroxyl H atom's acidity. The presence of water and MeOH ligands reduces the PA of** 1A**, the latter having the least effect. Like the case with IP, the presence of H_2_O ligands reduces the PA of** 2A**, while the presence of MeOH ligands increases that PA. The presence of water and MeOH ligands is observed to increase the PA of** 3A**, with the latter having the greatest effect.

The hydrogen of the O-H substituent is more acidic that that of the O_1_-H group of all L_2_ complexes, since the PA^*∗*^ and PAFE^*∗*^ of the former are lower than PA and PAFE of the latter, as shown in Table 4. It can be seen from Table 4 that the values of PAFE and PA for the chelates and complexes follow the same trend even though the PAFEs are lower than PAs. In fact, the differences between these PAs and PAFEs for the molecules studied range from 104 to 228 kJ/mol for** 1A** and** 3C,** respectively. However, both the PAs and PAFEs are higher than the PDEs and PDFEs, respectively, due to the high reactivity of the cationic radicals. As evidenced in Table 4, the values of ETE and ETFEs are, respectively, lower than those of IP and IPFEs.

#### 3.4.4. Thermodynamically Preferred Mechanism

**Figure 2:**
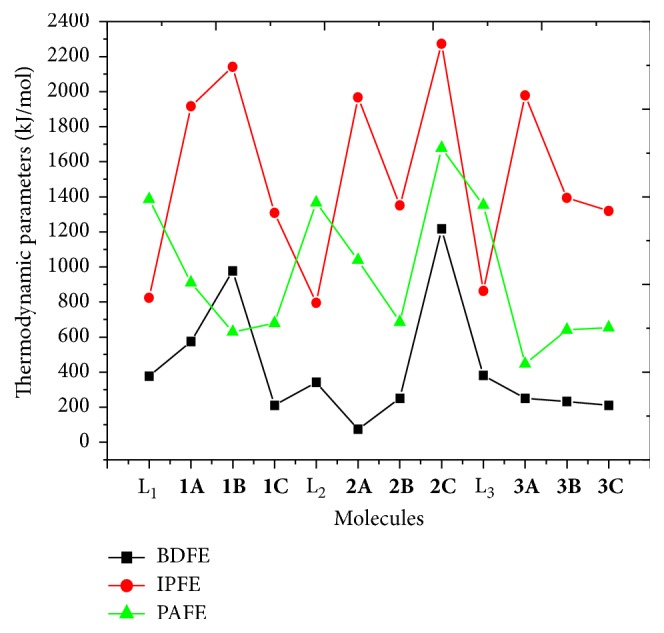
Superposition of BDFE, IPFE, and PAFE of juglone, its derivatives, and their complexes.

In order to select the thermodynamically preferred mechanism among the antioxidant mechanisms studied, the free energies of the first step of each mechanism have been compared. To facilitate this process, free energies for all the molecules investigated were plotted on the same axes as shown in Figure 2.

It is clear from this figure that the preferred mechanism for all the ligands as well as their chelates and complexes is the HAT, since each of these compounds exhibits the lowest free energy for this process. In the case of** 1B**, the SPLET is the most preferred mechanism since the free energy for PA is lower than that for direct HAT. While the second preferred antioxidant mechanism for the ligands is SETPT, that for the chelates and complexes is SPLET. In the case of** 1B**, the second preferred mechanism is HAT.

#### 3.4.5. Spin Density Analysis

**Figure 3:**
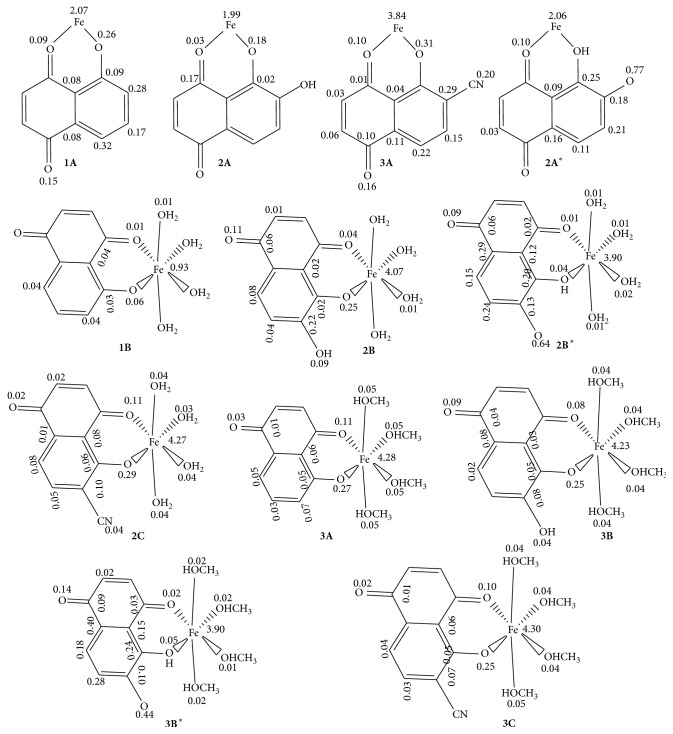
Mulliken spin densities of the radicals of the chelates and complexes studied.

The spin density is the most important parameter that correlates with the AOA of antioxidants [41]. It characterizes the stability of free radicals, since the energy of a free radical can be efficiently decreased if the unpaired electrons are highly delocalized through the conjugated system after hydrogen abstraction [5]. In Figure 3 the Mulliken spin density values on selected atoms of the chelates and complexes studied are presented.

Analyses of the structures in this figure have revealed a huge concentration on Fe(II) and the oxygen atom from which the H atom transferred is abstracted. Among the radicals formed from the chelates, that of** 2A**, the chelate with the lowest BDE (111 kJ/mol), is found to have the lowest spin density value on the central metal (1.99). On the other hand, the complex** 1B,** among all the compounds studied, has shown the lowest spin density value on the central metal (0.93). As a general observation for the rest of the complexes, the spin densities on the central Fe^2+^ ions are in the range 3.90–4.30. The spin density delocalization in all molecules studies has been greatly contributed by the carbon atoms in the two rings of juglone and its derivatives. As such, these carbon atoms significantly contribute to the stabilization of the radicals.

### 3.5. NBO Analysis

Natural Bond Orbital (NBO) refers to a suite of mathematical algorithms for analyzing electronic wave functions in terms of localized Lewis-like chemical bonds [37, 38]. In the current study, the strength of metal-ligand interactions has been estimated by means of the second-order perturbation theory, as applied in NBO analysis. For each chelate and complex, the stabilization energy or second-order perturbation energy, *E* ^(2)^ associated with the delocalization from *i* → *j* ((*i*) and (*j*) being the donor and acceptor orbitals, resp.), was estimated using

(16) $$\begin{array}{c} E^{\left(2\right)}=-q_{i}\frac{\left(F_{ij}\right)}{\varepsilon_{j}-\varepsilon_{i}}. \end{array}$$

Here, *q*_*i*_ is the orbital occupancy, *ε*_*i*_, *ε*_*j*_ are diagonal elements, and *F*_*ij*_ is the off-diagonal NBO Fock matrix element. Values of *E* ^(2)^ are proportional to the intensities of NBO interactions, and the greater the electron donating tendency from donor to acceptor NBOs, the larger the *E* ^(2)^ values and the more intensive the interaction between the electron donors and the electron acceptors [42, 43].

In Table 5, the values of *E* ^(2)^ (greater than 5 kJ/mol) for the ligand-metal charge transfer are reported, as well as the NPA charges of the central metal ions. It is worth noting that the values of *E* ^(2)^ are lower than 3 kJ/mol for metal-ligand charge transfer, signifying that these interactions are very weak and therefore ignored in this paper.

It can be observed from *E* ^(2)^ values in Table 5 that, with the exception of** 1A**, the interactions between the lone pairs on O_2_ and the antibonding LP^*∗*^ on the metal ion are stronger than those between the lone pairs on O_1_ and the antibonding LP^*∗*^ on Fe. In addition, the presence of water and methanol ligands which also interact strongly with Fe strengthens these ligand-metal interactions, thus accounting for the increment in the absolute values of the interaction energies as previously observed in Section 3.2 (Table 2). Among the compounds investigated, the strongest NBO interaction (that with the highest energy) is LP(2) O_1_ → LP^*∗*^(4) Fe in** 1A**, with a stabilization energy of 71.83 kJ/mol.

The atomic charges on Fe (Table 5) in the molecules under investigation have shown that ligand-metal charge transfer is effective, since the metal's formal charge of +2 now lies between 1.17 and 1.66. The highest atomic charge on Fe (1.66) is obtained in** 3A**, while the lowest value (1.17) is found in** 1B** and** 3B**.

### 3.6. Analysis of Metal-Ligand Bonds by the QTAIM

QTAIM is nowadays a strong tool used by quantum chemists to analyze the nature and strength of weak interactions [38]. In this work, the analysis of electron density (*ρ*(*r*)) and its Laplacian (∇^2^ *ρ*(*r*)) for the metal-ligand bonds has been performed by the means of the Bader QTAIM, as implemented in multiwfn. According to this theory, the sign of the Laplacian of the electron density (∇^2^ *ρ*(*r*)) at a bond critical point reveals whether charge is concentrated as in covalent bond interactions (∇^2^ *ρ*(*r*) < 0) or depleted as in closed shell (electrostatic) interactions (∇^2^ *ρ*(*r*) > 0) [41]. Specifically, for nonpolar and weakly polar covalent bonds ∇^2^ *ρ*(*r*) < 0 and −*G*(*r*)/*v*(*r*) < 1. For intermediate interactions we have ∇^2^ *ρ*(*r*) > 0, but −*G*(*r*)/*v*(*r*) < 1; and for closed shell interactions ∇^2^ *ρ*(*r*) > 0 and −*G*(*r*)/*v*(*r*) > 1. Here, *G*(*r*) is the kinetic energy density at the critical point (always positive), while *v*(*r*) is the potential energy density at the critical point (always negative) by definition [44]. The values of *ρ*(*r*), ∇^2^ *ρ*(*r*), and −*G*(*r*)/*v*(*r*) for all the metal-ligand bonds, as well as for the O-H bonds, are presented in Table 6.

From Table 6, it can be seen that each Fe-O bond has a positive ∇^2^ *ρ*(*r*) value except that of the O_1_-Fe bond of** 1A** which is negative (−0.108). This implies that the said O_1_-Fe bond is covalent in nature, while the rest of the Fe-O bonds are noncovalent. The covalent character of the O_1_-Fe bond of** 1A** provides a probable explanation to the fact that the perturbation energy (*E* ^(2)^ = 71.83 kJ/mol) corresponding to the interaction between O_1 _and Fe is the largest among the metal-ligand interactions.

The values of −*G*(*r*)/*v*(*r*) for the O_i_-Fe (*i* = 1 and 2) bonds are all less than unity (except for the O_1_-Fe bond of** 1B** which is a weak or closed shell interaction), meaning that these bonds are intermediate type interactions. Based on the values of −*G*(*r*)/*v*(*r*), it can be concluded that the X_*i*_-Fe (*i* = 1, 2, 3 and 4) bonds of** 1C**,** 2B**,** 2C**,** 3B**, and** 3C** (except the weak X_1_-Fe interaction of** 3C**) are also intermediate type interactions. On the contrary, X_*i*_-Fe (*i* = 1, 2, 3 and 4) bonds of** 1B** result from weak interactions. The negative values of ∇^2^ *ρ*(*r*) and the low values of −*G*(*r*)/*v*(*r*) as presented in Table 6 have clearly shown that the O-H bonds are all covalent.

**Figure 4:**
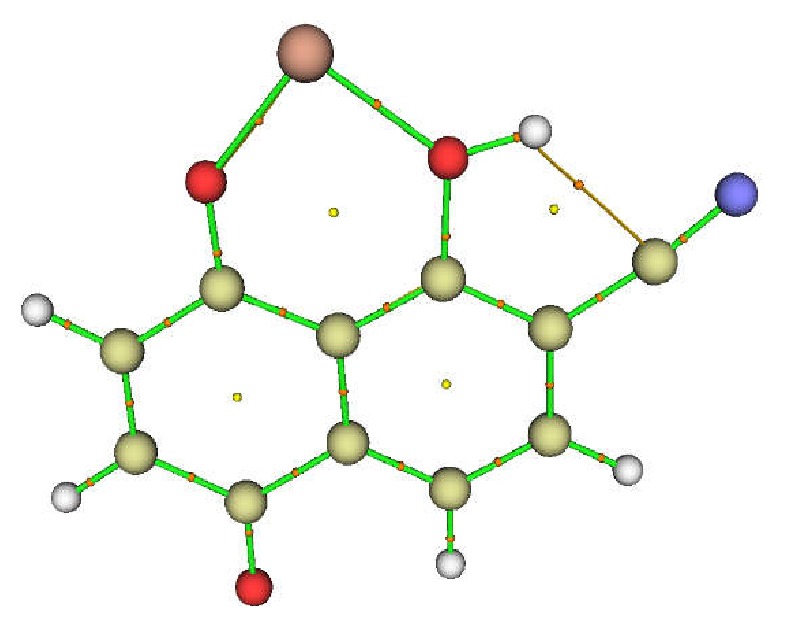
Molecular graph of** 3A**: bond critical points (small red spheres), ring critical points (small yellow sphere), bond paths (green lines), and O-H⋯CN VDW interaction (orange line).

The topological analyses of the complexes of L_2 _have confirmed the hydrogen bond O_1_-H⋯O earlier observed in their optimized geometries (Figure 1). Surprisingly, the topological analysis of the complexes of L_3_ revealed the existence of a Van der Waals (VDW) interaction between the hydrogen atom of the O-H group and the carbon atom of the -CN group as illustrated in Figure 4. This VDW interaction justifies the large O_1_-H bond length for the complexes of L_3_ (see Table 3). Hence, the VDW interaction has caused the elongation and weakening of the O-H, resulting in the relatively low BDE values observed for these complexes.

## 4. Conclusion

A DFT/B3LYP study has been performed herein in order to determine the chelating ability of neutral juglone and two of its derivatives towards Fe^2+^. The AOA of the resulting chelates and mixed ligand complexes with either H_2_O or CH_3_OH as another ligand were also evaluated. Precisely, the Gibbs free energies, the binding energies, and NBO analysis were used to evaluate the ability of L_1_, L_2_, and L_3_ to chelate Fe(II) ions. QTAIM was also used to investigate the degree of covalency of the metal-ligand bonds. The usual thermodynamic parameters BDE, IP, PDE, PA, and ETE and their free energies used in predicting the radical scavenging activities of antioxidants were used to study the effect of Fe^2+^ chelation on the AOA of the ligands, chelates, and complexes.

The negative values of Δ*G* and Δ*E*_int_ have indicated that the three ligands are capable of chelating the ferrous state of iron. These parameters have been relatively modified by the presence of H_2_O or CH_3_OH ligands in the complexes. NBO analysis has showed that metal-ligand bonds in the chelates and complexes result from metal-to-ligand charge transfer. Moreover, the second-order perturbation energy corresponding to the O_2_-Fe bonds of all the molecules with the exception of** 1A** have been found to be higher than those of the O_1_-Fe bond indicating that the former bonds are relatively stronger than the latter. QTAIM showed that, apart from the O_1_-Fe bond in** 1A** which is purely covalent, nearly all the O_1_-Fe and O_2_-Fe bonds of the rest of the molecules are intermediate type interactions. Also, the X_*i*_-Fe (*i* = 1, 2, 3 and 4) bonds in** 1C**,** 2B**,** 2C**,** 3B**, and** 3C** (except the weak X_1_-Fe interaction in** 3C**) are intermediate type interactions, whereas the X_*i*_-Fe (*i* = 1, 2, 3 and 4) bonds of** 1B** result from weak interactions.

Our results also revealed that while the chelation of Fe(II) reduces the BDE of L_2_ and L_3_, that of L_1 _instead increases. Furthermore, the reduction in the O_1_-H bond length in** 1B** and the presence of H_2_O ligands lead to an increase in the BDE of the chelate, while the addition of four CH_3_OH ligands to** 1A** instead decreases its BDE. The presence of H_2_O and CH_3_OH ligands increases the BDE of** 2A** but decreases that of** 3A**. QTAIM has revealed a VDW interaction between the H atom of the hydroxyl group of L_3_ and the C atom of its CN group which elongates and weakens the O-H bond of the complexes of L_3_, conferring them relatively low BDEs. On the other hand, BDE of** 2B** has been found to be the lowest owing to the presence of a hydrogen bond which stabilizes its radical. The IPs of the three ligands have been found to increase following their chelation of Fe^2+^, an observation which agrees with those in the literature. With the exception of** 2C**, the PA values of all the complexes and chelates studied have been found to be lower than those of their corresponding ligands. Analyses of the free energies of the first step of each hydrogen transfer mechanism revealed that the direct HAT is the preferred mechanism of the radical scavenging activity of nearly all the molecules studied in the present work. Solvent effects are going to be addressed in a subsequent article.

## Figures and Tables

**Table 1:** Energies (Hartree) of the compounds related to their multiplicities obtained from B3LYP/6-31G(d)(Fe)U6-31+G(d,p)(E).

Spin multiplicity		Singlet	Quintet
[Fe(L_1_)]^2+^	**1A**	−1873.21293208	−1873.12426700
[Fe(L_1_)(OH_2_)_4_]^2+^	**1B**	−2179.23172776	−2178.98173479
[Fe(L_1_)(CH_3_OH)_4_]^2+^	**1C**	−2336.10729027	−2336.15725602
[Fe(L_2_)]^2+^	**2A**	−1948.45209999	−1948.33220631
[Fe(L_2_)(OH_2_)_4_]^2+^	**2B**	−2254.13720775	−2254.18434320
[FeL_2_(CH_3_OH)_4_]^2+^	**2C**	−2411.68291413	−2411.72799125
[Fe(L_3_)]^2+^	**3A**	−1965.22170297	−1965.31620465
[Fe(L_3_)(OH_2_)_4_]^2+^	**3B**	−2271.13120555	−2271.17906337
[Fe(L_3_)(CH_3_OH)_4_]^2+^	**3C**	−2428.30591545	−2428.35653764

**Table 2:** Binding energy (Δ*E*_int_), enthalpy (Δ*H*) and free energy change (Δ*G*) of formation of the complexes all in kJ/mol, obtained from B3LYP/6-31G(d)(Fe)U6-31+G(d,p)(E).

Molecule	Δ*H*	Δ*G*	Δ*E*_int_
**1A**	−1000,08	−963,77	−997,60
**1B**	−4332,08	−1500,67	−4319,69
**1C**	−6230,60	−774,45	−6218,19
**2A**	−8900,34	−986,77	−8897,86
**2B**	−11483,96	−791,41	−11471,56
**2C**	−15004,90	−1677,43	−14992,50
**3A**	−16395,51	−609,87	−16393,04
**3B**	−19323,20	−757,95	−19310,81
**3C**	−21880,49	−668,61	−21868,09

**Table 3:** Metal-ligand and O-H bond lengths (*Ǻ*) of the complexes obtained from B3LYP/6-31G(d)(Fe)U6-31+G(d,p)(E).

	O_1_-H	O_1_-Fe	O_2_-Fe	X_1_-Fe	X_2_-Fe	X_3_-Fe	X_4_-Fe
**1A**	0.979	1.870	1.785	—	—	—	—
**1B**	0.971	1.982	1.898	2.029	2.021	2.023	2.024
**1C**	0.977	2.185	1.991	2.140	2.080	2.187	2.111
**2A**	0.993 0.972^*∗*^	1.863	1.796	—	—	—	—
**2B**	0.991 0.976^*∗*^	2.145	1.994	2.088	2.145	2.140	2.139
**2C**	0.977 0.969^*∗*^	2.221	2.010	2.196	2.117	2.186	2.137
**3A**	0.997	1.963	1.854	—	—	—	—
**3B**	0.986	2.167	1.987	2.090	2.145	2.138	2.137
**3C**	0.984	2.188	1.994	2.143	2.075	2.176	2.102

**Table 4:** Values of BDE, BDFE, IP, IPFE, PDE, PDFE, PA, PAFE, ETE and ETFE of the compounds studied, all in kJ/mol obtained from B3LYP/6-31G(d)(Fe)U6-31+G(d,p)(E).

	BDE	BDFE	IP	IPFE	PDE	PDFE	PA	PAFE	ETE	ETFE

L_1_	417	377	834	824	899	864	1422	1386	312	309
**1A**	608	575	1919	1917	6	−23	1014	910	1041	1038
**1B**	1001	977	2132	2142	186	154	793	629	1710	1720
**1C**	233	211	1299	1309	251	222	898	679	897	904
L_2_	380 359^*∗*^	341 323^*∗*^	805	795	892	874	1377 1361^*∗*^	1366 1330^*∗*^	319 314^*∗*^	315 312^*∗*^
**2A**	111 798^*∗*^	74 765^*∗*^	1967	1967	−539	−574	1150 1008^*∗*^	1039 981^*∗*^	414 1107^*∗*^	407 1104^*∗*^
**2B**	271 358^*∗*^	250 327^*∗*^	1338	1351	251	218	868 658^*∗*^	685 636^*∗*^	928 1016^*∗*^	937 1009^*∗*^
**2C**	1240 1324^*∗*^	1217 1301^*∗*^	2255	2273	245	262	1904 1648^*∗*^	1678 1625^*∗*^	908 993^*∗*^	910 994^*∗*^
L_3_	421	381	874	863	864	828	1366	1353	371	368
**3A**	283	250	1833	1979	−233	−410	570	447	1176	1175
**3B**	248	232	1375	1394	191	157	829	641	953	963
**3C**	234	211	1308	1319	243	211	881	653	924	930

*∗* refers to the parameters related to the O-H substituent present on L_2_.

**Table 5:** NBO analysis of the metal-ligand bonds and NPA atomic charge of the central Fe in the chelates and complexes obtained from B3LYP/6-31G(d)(Fe)U6-31+G(d,p)(E).

	NPA atomic charge Fe (e)	Donor (*i*)	Acceptor (*j*)	*E* ^(2)^ (kJ/mol)	Donor (*i*)	Acceptor (*j*)	*E* ^(2)^ (kJ/mol)
**1A**	1.45	LP(2) O_1_	LP^*∗*^(4) Fe	71.83	LP(1) O_2_	LP^*∗*^(6) Fe	27.02
		LP(2) O_1_	LP^*∗*^(6) Fe	11.87	LP(2) O_2_	LP^*∗*^(5) Fe	12.16

**1B**	1.17	LP(2) O_1_	LP^*∗*^(4) Fe	69.90	LP(2) X_1_	LP^*∗*^(8) Fe	34.08
		LP(2) O_1_	LP^*∗*^(7) Fe	26.77	LP(2) X_2_	LP^*∗*^(7) Fe	38.23
		LP(2) O_2_	LP^*∗*^(5) Fe	25.54	LP(2) X_3_	LP^*∗*^(8) Fe	32.17
		LP(2) O_2_	LP^*∗*^(9) Fe	32.31	LP(2) X_4_	LP^*∗*^(5) Fe	32.58

**1C**	1.46	LP(2) O_1_	LP^*∗*^(6) Fe	13.53	LP(2) X_1_	LP^*∗*^(9) Fe	10.55
		LP(1) O_2_	LP^*∗*^(6) Fe	8.27	LP(2) X_2_	LP^*∗*^(8) Fe	10.22
		LP(2) O_2_	LP^*∗*^(7) Fe	7.15	LP(2) X_3_	LP^*∗*^(7) Fe	10.40
		LP(2) O_26_	LP^*∗*^(6) Fe	7.52	LP(2) X_4_	LP^*∗*^(9) Fe	11.09

**2A**	1.33	LP(2) O_2_	LP^*∗*^(4) Fe	68.47	LP(1) O_2_	LP^*∗*^(6) Fe	8.73
		LP(2) O_2_	LP^*∗*^(6) Fe	11.65	LP(1) O_1_	LP^*∗*^(6) Fe	27.90
		LP(1) O_2_	LP^*∗*^(6) Fe	9.44	LP(2) O_1_	LP^*∗*^(5) Fe	12.37

**2B**	1.43	LP(2) O_1_	LP^*∗*^(2) Fe	19.56	LP(2) X_1_	LP^*∗*^(7) Fe	8.42
		LP(2) O_1_	LP^*∗*^(4) Fe	8.28	LP(2) X_2_	LP^*∗*^(4) Fe	15.23
		LP(1) O_2_	LP^*∗*^(7) Fe	12.00	LP(2) X_3_	LP^*∗*^(5) Fe	13.71
		LP(1) O_2_	LP^*∗*^(2) Fe	7.84	LP(2) X_4_	LP^*∗*^(5) Fe	13.72

**2C**	1.51	LP(2) O_1_	LP^*∗*^(2) Fe	18.76	LP(2) X_1_	LP^*∗*^(7) Fe	10.24
		LP(2) O_1_	LP^*∗*^(4) Fe	11.33	LP(2) X_2_	LP^*∗*^(4) Fe	13.09
		LP(1) O_2_	LP^*∗*^(7) Fe	9.25	LP(2) X_3_	LP^*∗*^(5) Fe	13.71
		LP(1) O_2_	LP^*∗*^(2) Fe	10.44	LP(2) X_4_	LP^*∗*^(5) Fe	12.24

**3A**	1.66	LP(2) O_1_	LP^*∗*^(3) Fe	14.10	LP(1) O_2_	LP^*∗*^(4) Fe	7.86
		LP(1) O_2_	LP^*∗*^(2) Fe	25.40	—	—	—

**3B**	1.17	LP(2) O_2_	LP^*∗*^(2) Fe	19.41	LP(2) X_1_	LP^*∗*^(6) Fe	10.44
		LP(2) O_2_	LP^*∗*^(4) Fe	8.90	LP(2) X_2_	LP^*∗*^(4) Fe	15.45
		LP(1) O_1_	LP^*∗*^(7) Fe	13.02	LP(2) X_3_	LP^*∗*^(5) Fe	13.70
		LP(1) O_1_	LP^*∗*^(2) Fe	7.26	LP(2) X_4_	LP^*∗*^(5) Fe	13.82

**3C**	1.20	LP(2) O_2_	LP^*∗*^(2) Fe	14.45	LP(2) X_1_	LP^*∗*^(6) Fe	9.12
		LP(2) O_2_	LP^*∗*^(5) Fe	7.55	LP(2) X_2_	LP^*∗*^(5) Fe	9.53
		LP(1) O_1_	LP^*∗*^(2) Fe	7.74	LP(2) X_3_	LP^*∗*^(3) Fe	12.40
		LP(1) O_1_	LP^*∗*^(6) Fe	5.91	LP(2) X_4_	LP^*∗*^(2) Fe	7.93

**Table 6:** Topological analysis of the metal-ligand and O-H bonds of the complexes.

	Parameter	O_1_-H_1_	O_1_-Fe	O_2_-Fe	X_1_-Fe	X_2_-Fe	X_3_-Fe	X_4_-Fe
**1A**	*ρ*(*r*)	0.342	0.228	0.132	—	—	—	—
	∇^2^ *ρ*(*r*)	−0.211	−0.108	0.894	—	—	—	—
	−*G*(*r*)/*v*(*r*)	0.088	0.477	0.941	—	—	—	—

**1B**	*ρ*(*r*)	0.353	0.637	0.354	0.056	0.057	0.058	0.058
	∇^2^ *ρ*(*r*)	−2.123	0.541	0.061	0.436	0.454	0.440	0.446
	−*G*(*r*)/*v*(*r*)	0.097	1.071	0.506	1.061	1.069	1.056	1.060

**1C**	*ρ*(*r*)	0.327	0.048	0.075	0.054	0.062	0.049	0.058
	∇^2^ *ρ*(*r*)	−1.576	0.213	0.464	0.278	0.313	0.227	0.297
	−*G*(*r*)/*v*(*r*)	0.116	0.093	0.987	0.949	0.960	0.922	0.947

**2A**	*ρ*(*r*)	0.326	0.110	0.337	—	—	—	—
		0.348^*∗*^						
	∇^2^ *ρ*(*r*)	−2.022	0.699	0.025	—	—	—	—
		−2.214^*∗*^						
	−*G*(*r*)/*v*(*r*)	0.088	0.963	0.503	—	—	—	—
		0.093^*∗*^						

**2B**	*ρ*(*r*)	0.310	0.052	0.076	0.060	0.053	0.053	0.053
		0.388^*∗*^						
	∇^2^ *ρ*(*r*)	−1.148	0.258	0.441	0.328	0.282	0.283	0.263
		−16.76^*∗*^						
	−*G*(*r*)/*v*(*r*)	0.113	0.951	0.980	0.972	0.956	0.956	0.950
		0.004^*∗*^						

**2C**	*ρ*(*r*)	0.347	0.044	0.073	0.047	0.057	0.048	0.054
		0.355^*∗*^						
	∇^2^ *ρ*(*r*)	−2.089	0.182	0.419	0.211	0.267	0.224	0.268
		−2.128^*∗*^						
	−*G*(*r*)/*v*(*r*)	0.099	0.918	0.981	0.925	0.946	0.926	0.946
		0.099^*∗*^						

**3A**	*ρ*(*r*)	0.300	0.084	0.110	—	—	—	—
	∇^2^ *ρ*(*r*)	−1.431	0.481	0.730	—	—	—	—
	−*G*(*r*)/*v*(*r*)	0.115	0.981	0.963	—	—	—	—

**3B**	*ρ*(*r*)	0.316	0.050	0.077	0.060	0.053	0.054	0.053
	∇^2^ *ρ*(*r*)	−1.511	0.239	0.451	0.328	0.239	0.286	0.284
	−*G*(*r*)/*v*(*r*)	0.117	0.943	0.983	0.972	0.950	0.958	0.957

**3C**	*ρ*(*r*)	0.320	0.047	0.323	0.078	0.194	0.050	0.059
	∇^2^ *ρ*(*r*)	−1.532	0.211	−0.341	0.031	0.172	0.238	0.311
	−*G*(*r*)/*v*(*r*)	0.117	0.934	0.453	1.214	0.436	0.929	0.956

*∗* refers to the parameters related to the O-H substituent present on L_2_.
